# Body Mass Index, Left Ventricular Wall Stress, and NT-proBNP in Elderly Adults

**DOI:** 10.1016/j.jacadv.2025.102564

**Published:** 2026-01-21

**Authors:** Audrey White, Shi Huang, Vineet Agrawal, Katherine N. Bachmann, Evan L. Brittain, Debra D. Dixon, Leonie Dupuis, Erica Garner, Kelly Schlendorf, Thomas J. Wang, Deepak K. Gupta

**Affiliations:** a Department of Medicine, Vanderbilt University Medical Center, Nashville, Tennessee, USA; b Department of Biostatistics, Vanderbilt University School of Medicine, Nashville, Tennessee, USA; c Division of Cardiovascular Medicine, Vanderbilt University Medical Center, Nashville, Tennessee, USA; d Tennessee Valley Healthcare System, Department of Veterans Affairs, Nashville, Tennessee, USA; e Vanderbilt Translational and Clinical Cardiovascular Research Center, Vanderbilt University Medical Center, Nashville, Tennessee, USA; f Division of Diabetes, Endocrinology and Metabolism, Vanderbilt University Medical Center, Nashville, Tennessee, USA; g Department of Medicine, University of Texas-Southwestern Medical Center, Dallas, Texas, USA

**Keywords:** cardiac function, echocardiogram, left ventricular wall stress, natriuretic peptides, obesity

## Abstract

**Background:**

Obesity is a major risk factor for heart failure (HF). Natriuretic peptides (NPs) are cardioprotective hormones released in response to left ventricular (LV) wall stress (WS). Paradoxically, body mass index (BMI) and NP levels are inversely related, suggesting that the associations between BMI, WS, and NPs are not fully understood, particularly among individuals without HF.

**Objectives:**

The purpose of this study was to test the hypothesis that WS mediates, in part, the relationship between BMI and NP levels.

**Methods:**

In 4,444 Atherosclerosis Risk in Communities study participants without HF who underwent transthoracic echocardiography (median 75 years, BMI 27.7 kg/m^2^, 58% women, 18% Black), LV end-diastolic and end-systolic WS (DWS, SWS) were calculated from chamber dimensions, E/e’, and blood pressure. Mediation analysis was performed using the product of coefficients method adjusted for demographics, cardiovascular risk factors, cardiovascular disease, medications, creatinine, glucose, LV ejection fraction, and LV mass index.

**Results:**

BMI inversely associated with log-transformed N-terminal pro B-type natriuretic peptide (NT-proBNP) (β = −0.069 per 5 kg/m^2^; 95% CI: −0.096 to −0.042). BMI positively associated with DWS (β = 0.264 per 5 kg/m^2^; 95% CI: 0.073-0.453) but not SWS (β = −0.168 per 5 kg/m^2^; 95% CI: −0.708 to 0.403). DWS and SWS positively associated with log-transformed NT-proBNP (DWS: β = 0.011; 95% CI: 0.005-0.015; SWS: β = 0.004; 95% CI: 0.002-0.006). The association of BMI and NT-proBNP was partially mediated by DWS (mediation effect: 0.0028; 95% CI: 0.0006-0.0055) but not SWS (mediation effect: −0.0006; 95% CI: −0.0032 to 0.0015).

**Conclusions:**

Among older adults without HF, BMI positively associates with DWS, and DWS positively relates with NT-proBNP, which does not account for the net inverse relationship between BMI and NT-proBNP.

Higher body mass index (BMI) and obesity strongly associate with greater risk for incident heart failure (HF).^1^ The mechanisms by which higher BMI contributes to increased risk for HF are incompletely understood, but obesity cardiomyopathy may include subclinical abnormalities of cardiac structure and function, for example, hypertrophy, higher filling pressure, and worse global longitudinal systolic strain.^2^, ^3^, ^4^, ^5^ The aforementioned cardiac abnormalities typically associate with higher circulating natriuretic peptide (NP) levels as cardiomyocytes secrete these hormones to counteract increases in wall stress (WS), suggesting higher BMI should relate to greater WS and higher NP levels.^6^, ^7^, ^8^, ^9^ Paradoxically, an inverse association between BMI and circulating NP levels is evident among individuals with and without HF.^10^ One study of 12 individuals without HF undergoing bariatric surgery did not find a significant change in the NP to systolic WS relationship; however, this was limited to individuals with BMI >35 kg/m^2^ and did not include assessment of diastolic WS.^11^ As WS can be estimated noninvasively from transthoracic echocardiography (TTE), we used publicly available data from the ARIC (Atherosclerosis Risk in Communities) Study to examine the relationship of BMI, WS, and N-terminal pro B-type natriuretic peptide (NT-proBNP) among 4,444 elderly community dwelling individuals without prevalent HF.

## Methods

ARIC is a National Heart, Blood, and Lung Institute sponsored longitudinal observational study examining risk factors for cardiovascular disease in 4 U.S. communities (Forsyth County, North Carolina; Jackson, Mississippi; Washington County, Maryland; and Minneapolis, Minnesota). Institutional Review Board approval was obtained at each site, and participants provided written informed consent. The fifth ARIC visit took place between 2011 and 2013 and included ascertainment of medical history, biospecimen collection, and TTE. A total of 6,538 participated in the fifth ARIC visit, of whom 5,597 underwent TTE. Participants with a history of HF (n = 817), moderate or severe valvular disease (n = 85), those in whom WS could not be calculated (n = 94), NT-proBNP was not obtained (n = 135), or BMI was not ascertained (n = 22) were excluded, yielding an analytic cohort of 4,444 individuals.

### Echocardiography and wall stress

TTE was performed using a standardized protocol. Analysis and quantification of cardiac structure were completed at the Cardiovascular Imaging Core Lab at Brigham and Women’s Hospital, Boston, MA, as previously described by Shah et al.^12^ For the calculation of WS, wall thickness and left ventricular (LV) internal dimensions were obtained in the parasternal long-axis view, and hemodynamic data were derived from systemic blood pressure or from Doppler imaging of the mitral valve, mitral annulus, and aortic valve (AV). LV end-systolic and end-diastolic wall stress (SWS, DWS) were calculated using formulas previously validated against hemodynamic data^13^^,^^14^:

SWS = (0.334 × [SBP + peak AV gradient] × LVIDs)/(PWTs × [1 + PWTs/LVIDs]), where SBP = systolic blood pressure, LVIDs = LV internal diameter at end systole, and PWTs = posterior wall thickness in systole. Systolic blood pressure was measured from the brachial artery using a standard protocol. Aortic valve gradient was estimated from peak flow velocity using the simplified Bernoulli equation.

DWS = (0.334 × PCWP × LVIDd)/[PWTd × (1 + (PWTd/LVIDd)] where PCWP = pulmonary capillary wedge pressure, LVIDd = LV internal diameter at end diastole, and PWTd = posterior wall thickness in diastole. The simplified formula of 4 + E/e’ from early mitral inflow velocity (E) divided by the average of septal and lateral wall (e’) was used to estimate pulmonary capillary wedge pressure.^15^

### Demographics, anthropometrics, and clinical characteristics

BMI was calculated from weight and height. Prevalent hypertension, diabetes, smoking status, coronary artery disease, and atrial fibrillation/flutter were ascertained at visit 5 as previously described.^16^ Percentage body fat, fat mass, and lean body mass were determined from bioelectric impedance measured using the Tanita Body Composition Analyzer, TBF-300A. Hemoglobin A1c, fasting glucose, cholesterol, and triglycerides were measured using standard clinical assays. High-sensitivity cardiac troponin-T and NT-proBNP were measured from electrochemiluminescence immunoassay using the Roche Cobas e411 analyzer (Roche Diagnostics). The analytic measurement range for NT-proBNP was 5 to 35,000 pg/mL. Intraassay and interassay coefficients of variation for NT-proBNP were 2.7% and 3.2% at 175 pg/mL, 2.4% and 2.9% at 355 pg/mL, 1.9% and 2.6% at 1,068 pg/mL, and 1.8% and 2.3% at 4,962 pg/mL. C-reactive protein was measured in specimen from high-sensitivity immunonephelometric assay using the BNII nephelometer (Siemens Healthcare Diagnostics).

### Statistical analysis

For descriptive statistics, participants were categorized according to BMI <25 kg/m^2^, 25 to 30 kg/m^2^, and ≥30 kg/m^2^. Clinical characteristics and cardiac structure and function were reported as counts (percentages) for categorical variables and median (IQR) for continuous variables and compared between BMI categories and between quartiles of NT-proBNP using Pearson chi-squared or Kruskal-Wallis tests, as appropriate. To examine the relationships between BMI, DWS or SWS, and NT-proBNP, first the correlations between BMI, DWS or SWS, and NT-proBNP were assessed using Spearman correlation. Second, multivariable linear regression models were fitted with DWS or SWS, and NT-proBNP as outcomes, and BMI as the predictor. We visually examined regression model assumptions using histogram plots of outcomes, histogram plots of model residuals, QQ plots of model residuals, and model residuals vs fitted outcomes. Covariates were selected a priori based on literature search of cross-sectional correlates of WS and NP. The covariates in the multivariable model were age, sex, race, heart rate, systolic and diastolic blood pressure, diabetes, coronary heart disease, atrial fibrillation, hypertension, smoking, serum creatinine, serum glucose, hypertension medications, diabetes medications, LV ejection fraction (LVEF), and LV mass index. Potential multicollinearity was assessed by variance inflation factor. Due to skewed distributions of NT-proBNP, raw values were natural log-transformed prior to entry into regression models.

We hypothesized that 1) DWS and SWS mediated the relationship between BMI and NT-proBNP and 2) the mediation varied by BMI. For hypothesis testing, first the total effect between BMI and NT-proBNP was assessed by regressing NT-proBNP on BMI adjusted for covariates. Second, a parallel multiple mediators (ie, SWS and DWS) mediation was conducted to test mediation by DWS and SWS on the relationship between BMI and NT-proBNP (Central Illustration). In the mediation analysis, the total effect of BMI was decomposed into direct effect and 2 indirect effects (ie, mediation effects). To test for mediation effect, we used the “product of coefficients” test, which is based on the distribution of the indirect effect of predictor on outcome through the mediators.^17^ The product of the 2 pathways “a × b” is the indirect effect of the BMI on NT-proBNP through DWS and SWS. We tested whether the product “a × b” is statistically significant by calculating its 95% CI using bootstrap method, and then examining whether the 95% CI contains zero. In addition, to examine potential moderated mediation, that is, the mediation effect of WS varies by BMI levels, the interaction terms between BMI and WS were included in the mediation model to examine the effect on NT-proBNP. All analyses were conducted using R: a language and environment for statistical computing (version 4.4.0, R Foundation for Statistical Computing). Two-sided *P* values < 0.05 or 95% CI that did not contain zero were considered statistically significant for all analyses.

**Central Illustration:**
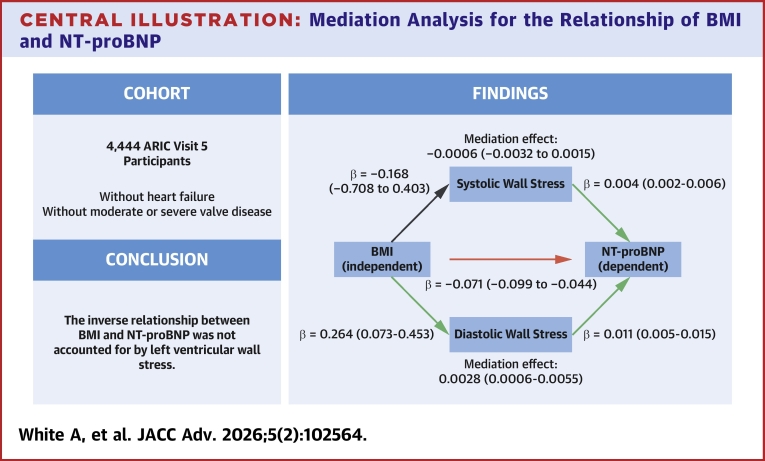
Mediation Analysis for the Relationship of BMI and NT-proBNP Mediation analysis for the effect of WS on the association between BMI and NT-proBNP among patients without heart failure in the ARIC (Atherosclerosis Risk In Communities) study. The total association between BMI and NT-proBNP is the sum of the direct association between BMI and NT-proBNP and the mediation by wall stress (DWS or SWS). The mediation effects were calculated from the product of regressing wall stress on BMI and regressing NT-proBNP on wall stress. Parentheses indicate 95% CIs. Covariates in the model were age, sex, race, heart rate, systolic and diastolic blood pressure, hypertension, atrial fibrillation, coronary heart disease, diabetes, serum creatinine, serum glucose, hypertension and diabetes medication use, LVEF, and LV mass index. ARIC = Atherosclerosis Risk in Communities; BMI = body mass index; DWS = left ventricular end-diastolic wall stress; LV = left ventricle; LVEF = left ventricular ejection fraction; NT-proBNP = N-terminal pro B-type natriuretic peptide; SWS = left ventricular end-systolic wall stress.

## Results

Table 1 displays the characteristics of the study cohort according to BMI. Overall, the study cohort had a median age of 75 years, 58% were female, and 18% identified as Black. Hypertension (71%) and diabetes (30%) were common, and CAD was prevalent in 10%. Obesity (BMI ≥30 kg/m^2^) was prevalent in 32%, overweight (BMI 25-<30 kg/m^2^) in 40%, normal weight (BMI 18.5-<25 kg/m^2^) in 26%, and underweight (BMI <18.5 kg/m^2^) in 1% of participants. Characteristics of the study cohort according to NT-proBNP quartiles are shown in Supplemental Table 1.

**Table 1:** Cohort Characteristics According to Body Mass Index

	Total (N = 4,444)	BMI <25 kg/m^2^ (n = 1,205)	BMI 25-30 kg/m^2^ (n = 1798)	BMI >30 kg/m^2^ (n = 1,441)	*P* Value
Age, y	75 [71, 79]	76 [72, 80]	75 [71, 79]	73 [71, 77]	<0.001
Female	2,586 (58%)	774 (64%)	962 (53%)	850 (59%)	<0.001
Race					<0.001
Black	780 (18%)	142 (12%)	292 (16%)	346 (24%)	
White	3,664 (82%)	1,063 (88%)	1,506 (84%)	1,095 (76%)	
BMI, kg/m^2^	27.7 [24.8, 31.1]	23.2 [21.5, 24.2]	27.4 [26.2, 28.7]	33.1 [31.3, 36.0]	<0.001
Weight, kg	76.8 [66.2, 88.5]	61.2 [55.4, 68.8]	75.3 [68.9, 82.9]	92.6 [83.6, 103.3]	<0.001
Fat percentage	34.8 [27.7, 41.4]	27.9 [22.3, 33.6]	34.7 [27.6, 40.2]	42.4 [35.3, 46.3]	<0.001
Fat mass, kg	26.2 [19.9, 33.7]	17.1 [14.1, 20.2]	25.6 [21.9, 28.9]	37.3 [32.3, 43.3]	<0.001
Lean body mass, kg	47.9 [41.6, 59.2]	41.1 [38.0, 52.9]	47.0 [41.7, 59.5]	53.0 [46.3, 64.2]	<0.001
Total body water, kg	35.1 [30.5, 43.3]	30.2 [27.8, 38.7]	34.4 [30.5, 43.6]	38.8 [33.9, 47.0]	<0.001
Waist hip ratio	0.95 [0.88, 0.99]	0.91 [0.85, 0.96]	0.95 [0.90, 0.99]	0.96 [0.90, 1.02]	<0.001
Heart rate, beats/min	64 [58, 72]	64 [58, 72]	64 [57, 71]	66 [59, 73]	<0.001
Systolic BP, mm Hg	129 [118, 141]	129 [117, 141]	129 [119, 140]	129 [118, 141]	0.76
Diastolic BP, mm Hg	66 [60, 73]	64 [57, 72]	66 [60, 73]	68 [62, 75]	<0.001
Hypertension	3,146 (71%)	704 (58%)	1,284 (71%)	1,158 (80%)	<0.001
Diabetes	1,112 (25%)	147 (12%)	425 (24%)	540 (38%)	<0.001
CAD	434 (10%)	105 (9%)	216 (12%)	113 (8%)	<0.001
Atrial fibrillation	205 (5%)	52 (4%)	86 (5%)	67 (5%)	0.88
Smoking					<0.001
Current	251 (6%)	101 (9%)	91 (6%)	59 (4%)	
Former	2077 (51%)	529 (48%)	838 (51%)	710 (54%)	
Never	1756 (43%)	471 (43%)	727 (44%)	558 (42%)	
Creatinine, mg/dL	0.91 [0.78, 1.07]	0.87 [0.75, 1.02]	0.93 [0.79, 1.08]	0.92 [0.79, 1.09]	<0.001
High-sensitivity troponin T, pg/mL	0.010 [0.007, 0.015]	0.010 [0.007, 0.014]	0.010 [0.007, 0.015]	0.011 [0.007, 0.016]	<0.001
NT-proBNP, pg/mL	122.7 [63.6, 232.4]	148.6 [78.9, 258.2]	114.2 [61.4, 228.9]	107.0 [56.3, 210.1]	<0.001
Triglycerides, mg/dL	112 [85, 150]	97 [75, 128]	114 [87, 154]	123 [93, 166]	<0.001
Low-density lipoprotein, mg/dL	103 [81, 126]	108 [87, 131]	102 [81, 126]	98 [76, 123]	<0.001
High-density lipoprotein, mg/dL	51 [43, 60]	58 [49, 69]	50 [42, 59]	46 [40, 55]	<0.001
Total cholesterol, mg/dL	180 [155, 209]	189 [163, 218]	179 [154, 206]	173 [150, 201]	<0.001
Fasting glucose, mg/dL	106 [97, 118]	100 [94, 110]	106 [98, 118]	111 [102, 126]	<0.001
Insulin, μU/mL	10.8 [7.2, 16.4]	7.2 [4.9, 10.1]	10.9 [7.6, 15.6]	15.5 [10.6, 22.6]	<0.001
C-reactive protein, mg/dL	1.91 [0.93, 4.07]	1.27 [0.65, 2.58]	1.88 [0.93, 3.81]	2.83 [1.42, 5.44]	<0.001
Hypertension medication use	2,141 (48%)	457 (38%)	883 (49%)	801 (56%)	<0.001
Diabetes medication use	767 (17%)	91 (8%)	281 (16%)	395 (27%)	<0.001
LV internal diameter in diastole, cm	4.34 [4.03, 4.70]	4.15 [3.93, 4.49]	4.34 [4.05, 4.67]	4.51 [4.18, 4.86]	<0.001
LV internal diameter in systole, cm	2.56 [2.28, 2.88]	2.46 [2.21, 2.74]	2.56 [2.28, 2.87]	2.67 [2.36, 2.98]	<0.001
Posterior wall thickness in diastole, cm	0.90 [0.84, 0.99]	0.86 [0.80, 0.92]	0.90 [0.85, 0.99]	0.95 [0.87, 1.04]	<0.001
Interventricular septal wall thickness in diastole, cm	1.01 [0.92, 1.13]	0.95 [0.87, 1.05]	1.01 [0.93, 1.13]	1.07 [0.96, 1.18]	<0.001
LV end-diastolic volume, mL	77.2 [63.6, 94.6]	69.7 [58.4, 86.5]	76.9 [63.9, 94.2]	83.4 [69.2, 102]	<0.001
LV end-systolic volume, mL	25.9 [20.4, 33.8]	23.4 [18.6, 30.8]	25.9 [20.3, 33.3]	28.3 [22.2, 36.4]	<0.001
LVEF, %	66 [62, 69]	66 [62, 70]	66 [63, 69]	66 [62, 69]	0.30
Relative wall thickness	0.42 [0.38, 0.46]	0.41 [0.37, 0.45]	0.42 [0.38, 0.46]	0.42 [0.38, 0.47]	<0.001
LV mass index, g/m^2^	75.2 [65.5, 87.2]	71.7 [62.7, 81.9]	75.6 [66.1, 87.4]	78.3 [67.6, 91.3]	<0.001
LV geometry					<0.001
Normal	1,015 (23%)	246 (20%)	456 (25%)	313 (22%)	
Concentric remodeling	723 (16%)	167 (14%)	327 (18%)	229 (16%)	
Concentric LVH	1,258 (28%)	326 (27%)	486 (27%)	446 (31%)	
Eccentric LVH	1,447 (33%)	466 (39%)	529 (29%)	452 (31%)	
Left atrial volume index, mL/m^2^	24.2 [20.0, 29.5]	23.4 [19.4, 28.9]	24.2 [20.1, 29.0]	24.9 [20.4, 30.1]	<0.001
E, cm/s	64 [54, 77]	64 [53, 77]	63 [54, 75]	66 [55, 79]	<0.001
A, cm/s	78 [67, 91]	75 [63, 87]	78 [67, 91]	81 [70, 93]	<0.001
E/A	0.8 [0.7, 1.0]	0.8 [0.7, 1.0]	0.8 [0.7, 0.9]	0.8 [0.7, 0.9]	<0.001
e’ lateral, cm/s	6.8 [5.6, 8.2]	7.0 [5.7, 8.4]	6.8 [5.7, 8.2]	6.7 [5.6, 8.2]	<0.001
e’ septal, cm/s	5.6 [4.7, 6.6]	5.8 [4.9, 6.9]	5.5 [4.7, 6.4]	5.5 [4.7, 6.5]	<0.001
e’ average, cm/s	6.3 [5.4, 7.3]	6.5 [5.5, 7.6]	6.2 [5.3, 7.3]	6.2 [5.4, 7.2]	<0.001
E/e’	10.2 [8.4, 12.5]	10.0 [8.2, 12.2]	10.1 [8.3, 12.4]	10.6 [8.7, 12.9]	<0.001
Peak longitudinal strain, %	−18.4 [−19.8, −16.7]	−18.5 [−20.0, −16.9]	−18.3 [−19.7, −16.7]	−18.2 [−19.7, −16.4]	0.002
TR peak vel, cm/s	235 [217, 254]	233 [215, 251]	235 [217, 252]	239 [221, 259]	<0.001
DWS, kdyne/cm^2^	18.9 [15.8, 22.5]	18.9 [15.8, 22.7]	18.7 [15.7, 22.0]	19.0 [15.8, 22.9]	0.10
SWS, kdyne/cm^2^	48.8 [39.3, 60.1]	49.9 [40.7, 60.8]	48.7 [39.0, 60.7]	47.6 [38.8, 58.9]	0.01

Values are n or median [IQR]. Significance testing performed using Kruskal-Wallis test for continuous variables or Pearson chi-squared test for categorical variables.

BP = blood pressure; BMI = body mass index; CAD = coronary artery disease; DWS = left ventricular end-diastolic wall stress; LV = left ventricle; LVEF = left ventricular ejection fraction; LVH = left ventricular hypertrophy; NT-proBNP = N-terminal pro B-type natriuretic peptide; SWS = left ventricular end-systolic wall stress; TR = tricuspid regurgitation velocity.

In unadjusted analysis, BMI and SWS inversely correlated (Spearman's rho = −0.049, *P* = 0.001). BMI and DWS were not significantly correlated (Spearman's rho = 0.011, *P* = 0.460). Both DWS and SWS positively correlated with NT-proBNP (Spearman's rho = 0.15; *P* < 0.001 and Spearman’s rho = 0.12; *P* < 0.0001, respectively). BMI inversely correlated with NT-proBNP (Spearman’s rho = −0.12; *P* < 0.001).

### Multivariable models

In multivariable-adjusted linear regression models, BMI positively associated with DWS (β = 0.264; 95% CI: 0.073-0.453, per 5 kg/m^2^ increase) which was adjusted for demographics, comorbidities, including diabetes, as well as blood pressure, creatinine, LVEF, and LV mass index (Table 2). In contrast, BMI was not significantly associated with SWS (β = −0.168; 95% CI: −0.708 to 0.403, per 5 kg/m^2^ increase).

**Table 2:** Left Ventricular End-Diastolic Wall Stress Partially Mediates the Association of BMI and NT-proBNP Among Participants of the Atherosclerosis Risk in Communities Study (N = 4,444)

Regression	Β (95% CI)	*P* Value
Mediator: DWS		
BMI – DWS (a1 path)	0.264 (0.073, 0.453)	0.006
DWS – ln(NT-proBNP) (b1 path)	0.011 (0.005, 0.015)	<0.001
Mediation effect (a1 × b1)	0.0028 (0.0006, 0.0055)	
Mediator: SWS		
BMI – SWS (a2 path)	−0.168 (−0.708, 0.403)	0.54
SWS – ln(NT-proBNP) (b2 path)	0.004 (0.002, 0.006)	<0.001
Mediation effect (a2 × b2)	−0.0006 (−0.0032, 0.0015)	
Direct effect		
BMI – ln(NT-proBNP) (c’ path)	−0.071 (−0.099, −0.044)	<0.001
Total effect		
BMI – ln(NT-proBNP) (c path)	−0.069 (−0.096, −0.042)	<0.001

Ordinary least squares regression estimates are from 4 separate models with natural log-transformed NT-proBNP as the outcome variable, BMI as the main predictor, and wall stress as a covariate or as the outcome variable. Beta coefficients correspond to a 1 kdyne/cm^2^ increase in wall stress or 5 kg/m^2^ increase in BMI. The mediation (a × b) was considered statistically significant if the CI did not include 0. Other covariates in the model were age, sex, race, heart rate, systolic blood pressure, diastolic blood pressure, hypertension, atrial fibrillation, coronary heart disease, diabetes, serum creatinine, serum glucose, hypertension medication use, diabetes medication use, LVEF, and LV mass index.

Abbreviations as in Table 1.

Both DWS and SWS positively associated with natural log-transformed NT-proBNP (DWS β = 0.011; 95% CI: 0.005-0.015; SWS β = 0.004; 95% CI: 0.002-0.006 per 1 kdyne/cm^2^ increase). These relationships were adjusted for demographics, comorbidities, blood pressure, LVEF, and LV mass index. The overall association between BMI and natural log-transformed NT-proBNP was inverse (β = −0.069; 95% CI: −0.096 to −0.042, per 5 kg/m^2^ increase).

### Mediation analysis

The associations of BMI to WS and WS to NT-proBNP (a and b paths, respectively) and mediation by WS are shown in Table 2 and Central Illustration. DWS partially mediated the BMI-NT-proBNP relationship in a positive direction (mediation effect: 0.0028; 95% CI: 0.0006-0.0053). This partial mediation was in opposite direction compared to the total effect; that is, inverse relationship between BMI and NT-proBNP. Unlike DWS, SWS was not a significant mediator (mediation effect: −0.0006; 95% CI: −0.0032 to 0.0015) of the relationship between BMI and NT-proBNP. Interaction terms between BMI and WS with the outcome of NT-proBNP were nonsignificant (DWS: p-interaction = 0.973; SWS: p-interaction = 0.606), and, therefore, not included in the mediation analysis. No significant interaction was found between BMI and sex in association with NT-proBNP (*P*-interaction = 0.325). Addition of serum triglycerides and C-reactive protein, as markers of insulin resistance and inflammation, respectively, to the model did not attenuate the association between DWS and NT-proBNP. Addition of waist hip ratio as a marker of central adiposity likewise did not attenuate the association of DWS and NT-proBNP.

### Fat mass

When fat mass and height were included in the models in place of BMI, there was a similar positive association between fat mass and DWS (β = 0.022; 95% CI: 0.003-0.042 per kg increase) and between DWS and NT-proBNP (β = 0.010; 95% CI: 0.005-0.016), and no significant association of fat mass and SWS (β = −0.021; 95% CI: −0.073 to 0.030). Similarly, DWS partially mediated the fat mass-NT-proBNP relationship in a positive direction (mediation effect: 0.0002; 95% CI: 0.00003-0.0005).

### Sensitivity analysis

Sensitivity analysis was performed following recalculation of pulmonary capillary wedge pressure as (1.29 × E/e’ lateral) + 1.9 or E/e’ alone with similar associations of BMI to DWS and DWS to NT-proBNP (data not shown).

## Discussion

In a relatively large cohort of community dwelling older adults without prevalent HF, we examined the relationships between BMI, LV WS, and circulating NT-proBNP levels. Our principal findings are: 1) higher BMI associates with greater diastolic but not systolic WS; 2) both diastolic and systolic WS positively associate with NT-proBNP levels; and 3) positive mediation by DWS does not account for the net inverse relationship between BMI and NT-proBNP.

The obesity epidemic over the past several decades negated previously observed reductions in cardiovascular disease and its associated mortality.^18^ The mechanisms by which higher BMI contributes to cardiovascular risk are incompletely understood. Prior studies examining the association of greater BMI on cardiac structure and function found remodeling including greater LV wall thickness and filling pressure,^5^^,^^19^ which should associate with higher NP levels, yet lower circulating NP levels have been found with greater BMI. This has raised consideration for obesity as a condition of NP insufficiency.^20^

The inverse relationship between BMI and circulating NP levels is not fully understood. Potential mechanisms underlying this BMI-NP relationship include less NP production and greater NP clearance. NPs are produced and released by cardiomyocytes to counteract greater WS. Insofar as higher BMI is a major risk for cardiovascular disease, including HF, it would be expected that greater BMI would raise WS. Few studies, however, have directly examined WS in association with BMI in individuals without prevalent HF.^4^^,^^11^^,^^21^ In the current analysis, we found among older adults without HF, BMI positively associated with DWS but not SWS. In contrast, 2 previous studies found no association between obesity duration and DWS and no difference in DWS between subjects with obesity and normal-weight controls.^4^^,^^21^ The differing results may be attributable to smaller sample sizes (N < 100), different formulas for calculation of DWS, and Tissue Doppler Imaging for estimation of left ventricular end diastolic pressure (LVEDP) as used in the present study. In both prior studies, intraventricular pressure was estimated from systemic arterial pressure. In the current study, LVEDP is estimated from E/e’, as previously validated in comparison to invasively measured LVEDP among patients with mild to severe obesity without HF.^22^ E/e’ has been found to underestimate pulmonary capillary wedge pressure in patients with severe obesity (BMI>35),^19^ which could have led to underestimation of DWS among participants with higher BMI.^19^ That we observed a significant positive association between BMI and DWS despite this potential limitation affirms the main finding of a positive mediation by DWS. Our findings in a community dwelling population using noninvasive echocardiography to estimate WS align with a study of 203 patients with or without HF clinically referred for cardiac catheterization, in which positive associations were found between BMI and LVEDP and LVEDP and NT-proBNP, although mediation analysis was not performed.^23^ Moreover, few studies have examined both DWS and SWS as we did in our analysis. While we found a significant association of BMI with DWS, this was not the case for SWS.

We also found among older adults without HF, DWS and SWS positively associated with NT-proBNP levels. These results are consistent with findings among patients with HF^8^^,^^9^ and align with the known physiology of the NP system; namely, cardiomyocytes produce and release NPs in response to greater WS as a counter-regulatory mechanism. NPs cause natriuresis and diuresis, which should lower filling pressures, a determinant of WS, and also exert antihypertrophic effects to lessen adverse cardiac remodeling.^24^^,^^25^ Collectively, that greater BMI associates with higher DWS, which in turn associates with greater NP levels, suggests higher BMI should associate with higher circulating NP levels, however, a net inverse relationship exists.

Mechanisms underlying the inverse association between BMI and NP levels remain unresolved. The aforementioned findings of a positive association between BMI and DWS suggest lower NP production does not solely explain the overall inverse BMI-NP relationship. Moreover, we did not find the relationship between WS and NT-proBNP varied by BMI. Together these data point to greater NP clearance as the predominant mechanism by which greater BMI relates to lower NP levels. Prior work by our group and others not only demonstrated adipocytes express the NP clearance receptor but also that the relative expression of NP clearance receptor to the cyclic guanine monophosphate generating receptor (natriuretic peptide receptor-A) increases with higher BMI.^26^^,^^27^ As an exploratory analysis, we accounted for fat mass and found that higher fat mass associated with higher DWS, yet there was an overall inverse association between fat mass and NT-proBNP. Additionally, the inverse association between BMI and NP persisted even with adjustment for fat mass. Our analysis also included adjustment for creatinine suggesting renally mediated NP clearance does not explain the inverse BMI-NP relationship. In the previously mentioned study of 12 individuals without HF who underwent bariatric surgery, BNP clearance rate was greater before compared with after weight loss, supporting that greater BMI associates with enhanced NP clearance.^11^

Immunoassay characteristics are also unlikely to fully explain the inverse BMI-NP relationship. In ARIC, NT-proBNP was measured using the proBNP II immunoassay (Roche Diagnostics). This assay may not detect glycosylated post-translational isoforms of NT-proBNP, which may be greater in individuals with obesity compared to those without obesity.^28^ While these assay characteristics could potentially explain those for NT-proBNP, inverse associations with BMI and BNP and atrial NP have also been found,^29^ suggesting the BMI and NP relationship is biologically rooted. Recent findings using a mass spectrometry-based assay for measurement of mature carboxy-terminal BNP and atrial NP suggest higher BMI also associates with lower levels of these NPs.^30^

### Study limitations

Strengths of our study include the large sample size, biracial community-based sample, and core lab quantification of LV structure and function. Limitations should also be noted. Noninvasive estimation of WS by TTE has been previously validated^14^; however, invasive hemodynamic measurements remain the gold standard for quantification of WS. Mitral annulus e’ velocity is less associated with left atrial pressure in the setting of impaired LV relaxation, which could impact DWS estimates.^31^ Although patients with HF were excluded from the analysis, the possibility of subclinical impairment of LV relaxation in the study cohort cannot be entirely excluded. All models were adjusted for cardiovascular risk factors, laboratory data, LV mass index, and LVEF; however, residual confounding cannot be excluded. The observational cross-sectional nature of the study limits inferences regarding causation between BMI, WS, and NPs. Our analysis was restricted to data obtained as part of the ARIC visit 5 study protocol, and so we were not able to examine biologically active isoforms of BNP. We cannot exclude differential effects of fat distribution and type, such as visceral vs subcutaneous fat and epicardial adipose tissue, which were not available for analysis.

## Conclusions

Among older adults without HF, BMI positively associates with DWS, and DWS positively relates with NT-proBNP, which does not account for the net inverse relationship between BMI and NT-proBNP.

## Funding support and author disclosures

This work was supported by the 10.13039/100000002National Institutes of Health (grant numbers R01HL153607, R01HL148661, R01HL154153, R38HL167237) and 10.13039/100000133Agency for Healthcare Research and Quality (grant number P30 HS029767). Dr Bachmann is currently an Amgen employee, but the work in this manuscript is not related to her employment at Amgen. All other authors have reported that they have no relationships relevant to the contents of this paper to disclose.

Perspectives **COMPETENCY IN MEDICAL KNOWLEDGE:** High BMI associates with higher LV diastolic WS in older adults without HF. The inverse relationship between BMI and NT-proBNP is not accounted for by WS.

**TRANSLATIONAL OUTLOOK:** The inverse relation of BMI and NT-proBNP was not explained by LV WS, which implies that WS is not the primary driving factor for NP insufficiency in obesity. Left ventricular WS is a readily quantifiable noninvasive parameter which can help further understanding of subclinical LV remodeling in obesity.
