# Supplementary material for: Body Mass Index, Left Ventricular Wall Stress, and NT-proBNP in Elderly Adults
Source: JACC Adv. 2026 Jan 21;5(2):102564. doi: 10.1016/j.jacadv.2025.102564 (PMC12859251; doi:10.1016/j.jacadv.2025.102564)
Supplement: Supplemental Table 1 [file mmc1.pdf]

**Table S1: Baseline Characteristics According to Quartiles of NT-proBNP**

| | Total<br>N=4444 | Quartile 1<br>< 63.6<br>N=1111 | Quartile 2<br>63.6 to <122.7<br>N=1111 | Quartile 3<br>122.7 to <232.5<br>N=1111 | Quartile 4<br>≥ 232.5<br>N=1111 | P-value |
|---|---|---|---|---|---|---|
| Age, years | 75 [71, 79] | 72 [70, 76] | 74 [71, 78] | 75 [72, 79] | 78 [73, 82] | <0.001 |
| Female | 2586 (58%) | 539 (48%) | 642 (58%) | 724 (65%) | 681 (61%) | <0.001 |
| Race | | | | | | <0.001 |
| Black | 780 (18%) | 325 (29%) | 200 (18%) | 151 (14%) | 103 (9%) | |
| White | 3664 (82%) | 786 (71%) | 911 (82%) | 960 (86%) | 1007 (91%) | |
| BMI, kg/$m^2$ | 27.7 [24.8, 31.1] | 28.7 [25.7, 32.0] | 28.0 [25.1, 31.2] | 27.2 [24.2, 30.9] | 27.1 [24.2, 30.5] | <0.001 |
| Weight, kg | 76.8 [66.2, 88.5] | 81.2 [70.3, 92.9] | 74.6 [67.3, 88.7] | 74.6 [64.4, 85.4] | 74.6 [63.5, 85.3] | <0.001 |
| Fat Percentage | 34.8 [27.7, 41.4] | 35.2 [28.4, 41.6] | 35.5 [27.9, 41.9] | 35.1 [28.5, 41.6] | 33.5 [26.3, 40.2] | <0.001 |
| Fat Mass, kg | 26.2 [19.9, 33.7] | 27.8 [21.8, 35.6] | 26.9 [20.5, 33.9] | 25.9 [19.4, 33.1] | 24.3 [18.3, 31.3] | <0.001 |
| Lean Body Mass, kg | 47.9 [41.6, 59.2] | 53.1 [43.1, 61.7] | 48.3 [41.8, 58.9] | 45.8 [40.8, 56.3] | 46.5 [41.2, 58.7] | <0.001 |
| Total Body Water, kg | 35.1 [30.5, 43.3] | 38.9 [31.6, 45.2] | 35.4 [30.6, 43.1] | 33.5 [29.9, 41.2] | 34.0 [30.2, 43.0] | <0.001 |
| Waist Hip Ratio | 0.95 [0.88, 0.99] | 0.95 [0.90, 0.99] | 0.95 [0.88, 0.99] | 0.94 [0.88, 0.98] | 0.94 [0.88, 0.99] | <0.001 |
| HR, bpm | 64 [58, 72] | 67 [60, 74] | 65 [58, 72] | 64 [57, 70] | 63 [56, 70] | <0.001 |
| Systolic BP, mmHg | 129 [118, 141] | 126 [117, 136] | 128 [118, 138] | 130 [119, 142] | 133 [120, 146] | <0.001 |
| Diastolic BP, mmHg | 66 [60, 73] | 68 [62, 74] | 66 [60, 73] | 65 [59, 72] | 65 [58, 73] | <0.001 |
| Hypertension | 3146 (71%) | 749 (68%) | 760 (69%) | 767 (70%) | 870 (79%) | <0.001 |

| | | | | | | |
|---|---|---|---|---|---|---|
| Diabetes | 1112 (25%) | 328 (30%) | 277 (25%) | 241 (22%) | 266 (24%) | <0.001 |
| CAD | 434 (10%) | 50 (5%) | 84 (8%) | 107 (10%) | 193 (18%) | <0.001 |
| Atrial Fibrillation | 205 (5%) | 16 (1%) | 26 (2%) | 34 (3%) | 129 (12%) | <0.001 |
| Smoking | | | | | | 0.263 |
| Current | 251 (6%) | 59 (6%) | 58 (6%) | 65 (6%) | 69 (7%) | |
| Former | 2077 (51%) | 553 (54%) | 496 (49%) | 506 (50%) | 522 (51%) | |
| Never | 1756 (43%) | 411 (40%) | 457 (45%) | 452 (44%) | 436 (43%) | |
| Creatinine, mg/dL | 0.91 [0.78, 1.07] | 0.90 [0.77, 1.06] | 0.89 [0.76, 1.03] | 0.90 [0.76, 1.04] | 0.96 [0.81, 1.19] | <0.001 |
| EGFR-creatinine | 71.4 [59.5, 83.1] | 77.0 [64.9, 86.5] | 73.7 [62.5, 84.1] | 71.1 [58.9, 82.5] | 64.2 [50.6, 76.3] | <0.001 |
| EGFR-cystatin C | 62.5 [49.9, 75.8] | 70.7 [58.8, 82.6] | 64.9 [53.9, 77.7] | 61.0 [49.6, 73.8] | 52.5 [39.9, 65.7] | <0.001 |
| hs-cTnT, pg/mL | 0.010 [0.007, 0.015] | 0.009 [0.006, 0.013] | 0.010 [0.007, 0.014] | 0.010 [0.007, 0.014] | 0.013 [0.009, 0.020] | <0.001 |
| Triglycerides, mg/dL | 112 [85, 150] | 115 [88, 156] | 111 [84, 145] | 112 [84, 155] | 106 [83, 145] | 0.005 |
| LDL, mg/dL | 103 [80, 126] | 104 [82, 126] | 104 [82, 127] | 104 [83, 128] | 98 [76, 119] | <0.001 |
| HDL, mg/dL | 51 [43, 60] | 48 [42, 57] | 51 [43, 61] | 53 [44, 63] | 51 [43, 60] | <0.001 |
| Total Cholesterol, mg/dL | 180 [155, 209] | 181 [156, 208] | 183 [156, 211] | 183 [159, 214] | 174 [148, 202] | <0.001 |
| Fasting Glucose, mg/dL | 106 [97, 118] | 108 [100, 122] | 106 [99, 118] | 104 [96, 116] | 103 [95, 116] | <0.001 |
| Insulin, µU/mL | 10.8 [7.2, 16.4] | 13.1 [8.6, 20.2] | 10.9 [7.3, 17.6] | 10.0 [6.9, 14.6] | 9.6 [6.1, 14.3] | <0.001 |
| CRP, mg/dL | 1.91 [0.93, 4.07] | 1.75 [0.86, 3.87] | 1.89 [0.93, 3.85] | 2.03 [0.96, 4.17] | 2.02 [0.98, 4.25] | 0.010 |

| | | | | | | |
|---|---|---|---|---|---|---|
| Hypertension medication | 2141 (48%) | 413 (37%) | 490 (44%) | 533 (48%) | 705 (63%) | <0.001 |
| Diabetes medication | 767 (17%) | 225 (20%) | 187 (17%) | 157 (14%) | 198 (18%) | 0.006 |
| LVIDd, cm | 4.34 [4.03, 4.70] | 4.32 [4.02, 4.65] | 4.34 [4.01, 4.66] | 4.32 [4.04, 4.67] | 4.41 [4.08, 4.81] | <0.001 |
| LVIDs, cm | 2.56 [2.28, 2.88] | 2.54 [2.26, 2.82] | 2.55 [2.28, 2.85] | 2.54 [2.26, 2.87] | 2.63 [2.33, 3.00] | <0.001 |
| PWTd, cm | 0.90 [0.84, 0.99] | 0.91 [0.85, 0.99] | 0.89 [0.84, 0.99] | 0.88 [0.83, 0.97] | 0.92 [0.85, 1.03] | <0.001 |
| IVSd, cm | 1.01 [0.92, 1.13] | 1.02 [0.93, 1.12] | 1.01 [0.92, 1.12] | 0.99 [0.90, 1.11] | 1.03 [0.93, 1.16] | <0.001 |
| LVEDV, mL | 77 [64, 95] | 79 [65, 96] | 76 [63, 93] | 75 [63, 91] | 79 [64, 98] | <0.001 |
| LVESV, mL | 26 [20, 34] | 27 [21, 34] | 25 [20, 33] | 25 [20, 33] | 27 [21, 35] | <0.001 |
| LVEF, % | 66 [62, 69] | 66 [63, 69] | 67 [63, 70] | 66 [63, 70] | 66 [61, 69] | <0.001 |
| Relative wall thickness | 0.42 [0.38, 0.46] | 0.42 [0.38, 0.47] | 0.42 [0.38, 0.47] | 0.41 [0.38, 0.46] | 0.42 [0.38, 0.47] | 0.024 |
| LV mass index, g/m$^2$ | 75.2 [65.5, 87.2] | 72.4 [63.9, 83.3] | 74.1 [64.1, 84.9] | 73.7 [65.3, 85.8] | 81.3 [70.0, 95.5] | <0.001 |
| LV geometry | | | | | | <0.001 |
| Normal | 1015 (23%) | 320 (29%) | 270 (24%) | 224 (20%) | 201 (18%) | |
| Concentric remod | 723 (16%) | 245 (22%) | 176 (16%) | 145 (13%) | 157 (14%) | |
| Concentric LVH | 1258 (28%) | 267 (24%) | 334 (30%) | 308 (28%) | 349 (32%) | |
| Eccentric LVH | 1447 (33%) | 279 (25%) | 331 (30%) | 434 (39%) | 403 (36%) | |
| LAVI, mL/m$^2$ | 24.2 [20.0, 29.5] | 22.0 [18.2, 25.9] | 22.9 [19.2, 27.3] | 24.5 [20.5, 29.3] | 28.9 [23.8, 35.3] | <0.001 |
| E, cm/s | 64 [54, 77] | 61 [52, 71] | 62 [53, 74] | 65 [54, 77] | 70 [57, 86] | <0.001 |
| A, cm/s | 78 [67, 91] | 78 [67, 89] | 79 [68, 91] | 79 [67, 91] | 79 [66, 92] | 0.65 |

| | | | | | | |
|---|---|---|---|---|---|---|
| E/A | 0.8 [0.7, 1.0] | 0.8 [0.7, 0.9] | 0.8 [0.7, 0.9] | 0.8 [0.7, 1.0] | 0.9 [0.7, 1.1] | <0.001 |
| e’ lateral, cm/s | 6.8 [5.6, 8.2] | 6.9 [5.7, 8.3] | 6.8 [5.6, 8.1] | 6.8 [5.6, 8.2] | 6.9 [5.6, 8.3] | 0.46 |
| e’ septal, cm/s | 5.6 [4.7, 6.6] | 5.6 [4.8, 6.6] | 5.6 [4.8, 6.6] | 5.6 [4.7, 6.6] | 5.5 [4.6, 6.6] | 0.086 |
| e’ average, cm/s | 6.3 [5.4, 7.3] | 6.4 [5.5, 7.3] | 6.3 [5.4, 7.3] | 6.2 [5.4, 7.3] | 6.3 [5.3, 7.3] | 0.35 |
| E/e’ | 10.2 [8.4, 12.5] | 9.7 [8.2, 11.7] | 10.0 [8.3, 12.2] | 10.3 [8.5, 12.6] | 11.0 [9.0, 13.8] | <0.001 |
| Peak longitudinal strain, % | -18.4 [-19.8, -16.7] | -18.1 [-19.6, -16.6] | -18.6 [-19.9, -17.0] | -18.5 [-20.0, -16.9] | -18.1 [-19.7, -16.1] | <0.001 |
| TR peak vel, cm/s | 235 [217, 254] | 227 [211, 247] | 234 [216, 250] | 236 [218, 252] | 244 [225, 265] | <0.001 |
| DWS, kdyne/cm$^2$ | 18.9 [15.8, 22.5] | 17.9 [15.2, 21.3] | 18.4 [15.6, 21.8] | 19.2 [16.2, 22.7] | 20.0 [16.4, 24.7] | <0.001 |
| SWS, kdyne/cm$^2$ | 48.8 [39.3, 60.1] | 46.1 [37.9, 56.1] | 48.7 [39.0, 58.8] | 49.6 [40.0, 61.3] | 51.0 [40.5, 64.4] | <0.001 |

Data presented as N (percentage) or median [IQR]. Significance testing performed using Kruskal-Wallis test for continuous variables or Pearson chi-squared test for categorical variables.

BMI = body mass index, BP = blood pressure, CAD = coronary artery disease, CPAP= continuous positive airway pressure, CRP = C-reactive protein, DWS= left ventricular end diastolic wall stress, HR = heart rate, hs-cTnT = high sensitivity cardiac Troponin T, eGFR= estimated glomerular filtration rate, IVSd= interventricular septal wall thickness in diastole, LAVI= left atrial volume index, LV = left ventricle, LVEDV= left ventricular end diastolic volume, LVESV= left ventricular end systolic volume, LVIDd= left ventricular internal diameter in diastole, LVIDs= left ventricular internal diameter in systole, LVH= left ventricular hypertrophy, NT-proBNP= N- terminal pro B-type natriuretic peptide, PWTd= posterior wall thickness in diastole, RWT= relative wall thickness, SWS= left ventricular end systolic wall stress, TR peak vel: tricuspid regurgitation peak velocity.
